# Sintering of Fine Particles in Suspension Plasma Sprayed Coatings

**DOI:** 10.3390/ma3073845

**Published:** 2010-07-01

**Authors:** Leszek Latka, Sergey B. Goryachev, Stefan Kozerski, Lech Pawlowski

**Affiliations:** 1 Service of Thermal Spraying at ENSCL, BP 90108, 59650 Villeneuve d’Ascq, France; E-Mail: leszek.latka@o2.pl (L.L.); 2 Department of Welding at Faculty of Mechanics, Wroclaw University of Technology, 50-371 Wroclaw, Poland; E-Mail: stefan.kozerski@pwr.wroc.pl (S.K.); 3 Université Lille Nord de France, F-59000 Lille, France; E-Mail: serguei.goriatchev@ensc-lille.fr (S.B.G.); 4 USTL, UDSMM, F-59650 Villeneuve d’Ascq, France; 5 CNRS, EAC 8207, F-59650 Villeneuve d’Ascq, France

**Keywords:** suspension plasma spraying, coatings grow up, hydroxyapatite coatings, sintering

## Abstract

Suspension plasma spraying is a process that enables the production of finely grained nanometric or submicrometric coatings. The suspensions are formulated with the use of fine powder particles in water or alcohol with some additives. Subsequently, the suspension is injected into plasma jet and the liquid additives evaporate. The remaining fine solids are molten and subsequently agglomerate or remain solid, depending on their trajectory in the plasma jet. The coating’s microstructure results from these two groups of particles arriving on a substrate or previously deposited coating. Previous experimental studies carried out for plasma sprayed titanium oxide and hydroxyapatite coatings enabled us to observe either a finely grained microstructure or, when a different suspension injection mode was used, to distinguish two zones in the microstructure. These two zones correspond to the dense zone formed from well molten particles, and the agglomerated zone formed from fine solid particles that arrive on the substrate in a solid state. The present paper focuses on the experimental and theoretical analysis of the formation process of the agglomerated zone. The experimental section establishes the heat flux supplied to the coating during deposition. In order to achieve this, calorimetric measurements were made by applying experimental conditions simulating the real coatings’ growth. The heat flux was measured to be in the range from 0.08 to 0.5 MW/m^2^, depending on the experimental conditions. The theoretical section analyzes the sintering during the coating’s growth, which concerns the fine particles arriving on the substrate in the solid state. The models of volume, grain boundary and surface diffusion were analyzed and adapted to the size and chemistry of the grains, temperature and time scales corresponding to the suspension plasma spraying conditions. The model of surface diffusion was found to best describe the sintering during suspension plasma spraying. The formation of necks having the relative size equal to 10% of particle diameter was found to be possible during the thermal cycles occurring at the coatings’ deposition. Transmission electron microscopic observations of the agglomerated zone hydroxyapatite coating confirm the sintering of some of the fine grains.

## 1. Introduction 

**Figure 1:**
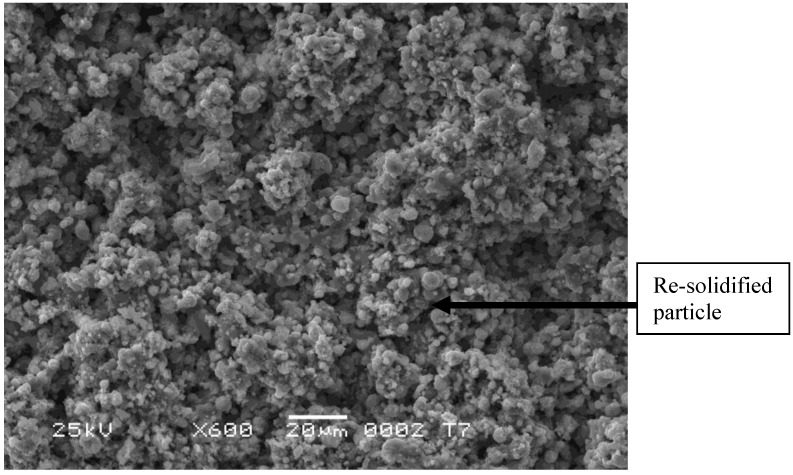
SEM (secondary electrons) micrograph of titanium oxide (TiO_2_) coatings suspension plasma sprayed using an external atomizing injector.

Suspension thermal spraying is, together with solution thermal spraying, a new process that enables production of finely grained nanometric or sub-micrometric coatings [1]. The suspensions are formulated usually with the use of fine powder particles in water or alcohol with some additives. Subsequently, the suspension can be injected as a continuous stream or atomized liquid into the plasma jet and the liquid additives evaporate. The microstructure of coatings depends strongly on the mode of suspension injection. The coatings sprayed using atomizing injectors are generally finely grained such as ZrO_2,_ stabilized with 8 wt % Y_2_O_3_ coatings as described in [2], or TiO_2_ coatings as described in [3] and shown in Figure 1.

**Figure 2:**
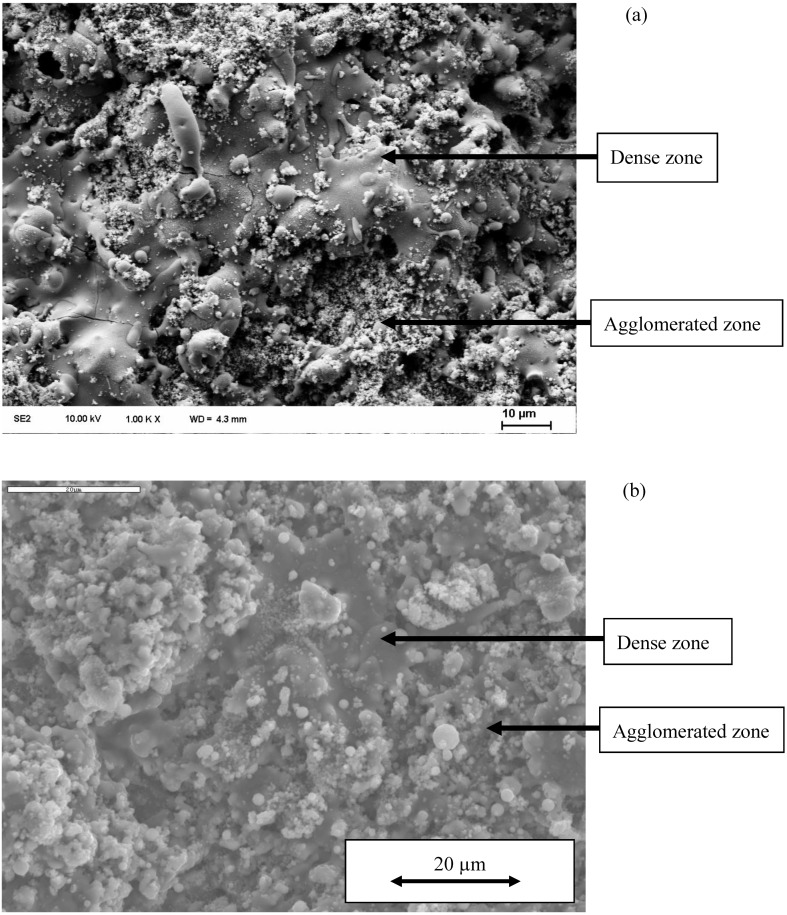
SEM (secondary electrons) micrographs of the coatings suspension plasma sprayed using internal, radial continuous-stream injector (a) hydroxyapatite (b) titanium oxide.

Similar microstructure is exhibited by the coatings plasma sprayed using a continuous-stream external injector (see e.g., Al_2_O_3_ coatings described in [4]). On the other hand, the processing with the use of plasma torches having internal injection results in coatings that have different microstructure. The axial internal suspension injection enabled Waldbillig and Kesler [5] to obtain ZrO_2_ + 20 wt % Y_2_O_3_ coatings having large, well molten grains with the pores characteristic for conventional coarse powder plasma spraying. An intermediate microstructure was observed when processing with internal radial injection [6,7,8]. The previous experimental studies carried out in our laboratory using the *SG 100* plasma spray torch from *Praxair* to spray Ca_5_(PO_4_)_3_OH (HA, hydroxyapatite) and TiO_2_ coatings with a continuous-stream, internal radial injection, enabled us to find out two zones of microstructure, which correspond to two groups of particles. These groups are: (i) well molten particles forming a dense zone, and (ii) fine solid particles that arrive on the substrate in a solid state, forming an agglomerated zone. The typical two zones microstructure is shown in Figure 2a and b.

The microstructure of the thermal spray coating results directly from the status of the grains when they arrive on the substrate or on the previously deposited coating. The large and well-deformed grains, having the form of splats, are visible in coatings sprayed using internal injectors. They agglomerate from fine initial particles in the plasma jet and are, later on, molten. These grains have their trajectory close to the hottest and fastest part of the plasma jet axis. They arrive on the substrate or on the previously deposited coating with a velocity that is sufficient for their deformation. On the contrary, the fine grains are close to the size of the solids used to formulate the suspension. They are sometimes spherical, which indicates that they were molten in the plasma jet. The ones with an irregular shape should have had their trajectory in the outer part the plasma jet. As the fine grains are not deformed, it is possible to deduce that they arrived on the substrate with low velocity.

The present study focuses on the analysis of the part of coatings’ microstructure formed by fine grains during suspension plasma spraying with the use of continuous stream injections. In particular, the thermophysical factors influencing their cohesion are analyzed. Firstly, the convective and radiative heat fluxes transferred to the growing coating is experimentally determined. The fluxes may cause the sintering of fine grains during the coating’s growth, which enhances the coating’s cohesion. On the other hand, the flux may generate residual stresses in the coatings, which can result in the formation of cracks. Different models of sintering are reviewed to determine which model is applicable for the time and temperature occurring during suspension plasma spraying. Finally, the calculations of the neck size between fine particles of Ca_5_(PO_4_)_3_OH and TiO_2_ are made for different temperatures and the results obtained are discussed in view of the microstructures observed in suspension sprayed deposits. 

## 2. Experimental Methods

**Figure 3:**
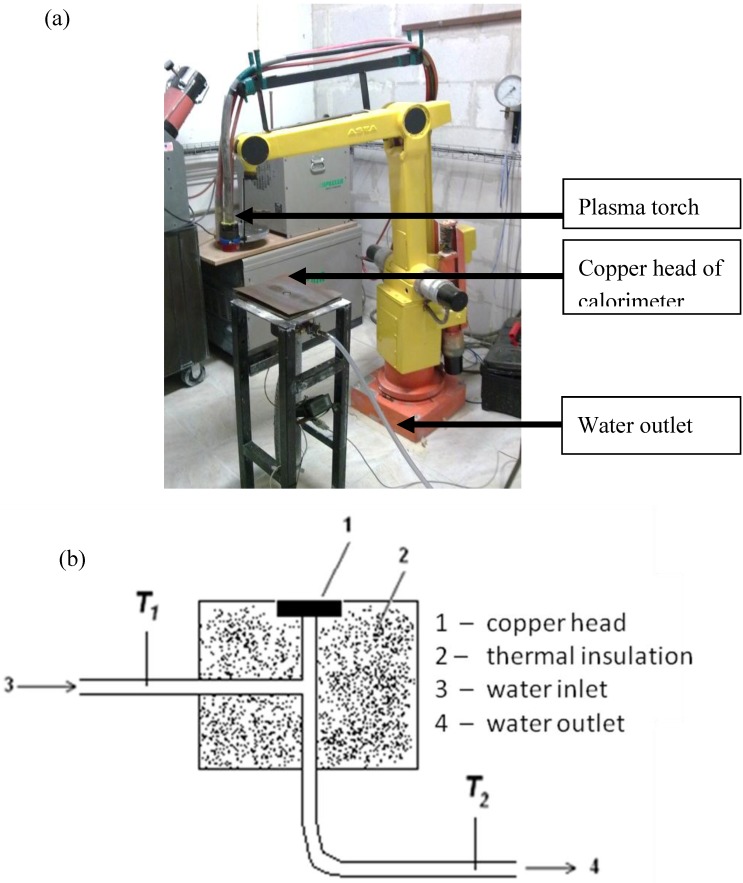
Experimental setup to determine the heat flux at suspension plasma spraying (a) a general overview (b) details of the calorimetric head.

The calorimetric measurements of heat flux simulated the experimental conditions during suspension plasma spraying of hydroxyapatite and titania coatings. The experimental setup for the calorimetric measurement of heat flux is shown in Figure 3. The copper head with a diameter of 25 mm was cooled down by water. The temperature of the water cooling the head was measured with a thermocouple. The heat flux was calculated with the use of the following equation:

(1) $$q=\frac{Q\rho c_{p}\Delta T}{S}$$

**Figure 4:**
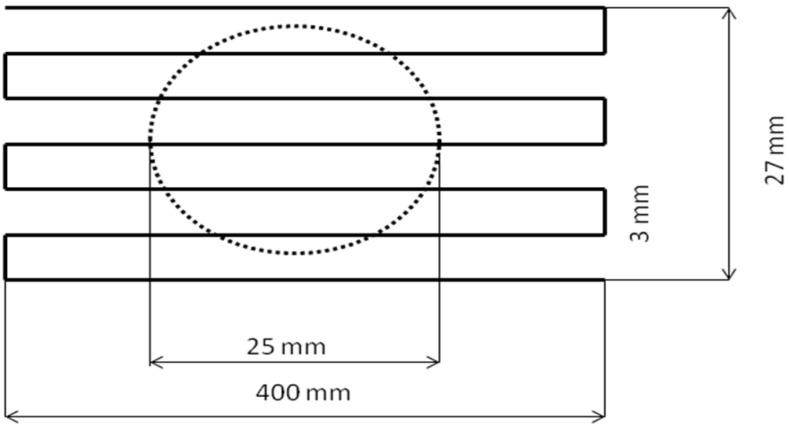
The trajectory of the torch in the calorimetric experiments.

In the experiments, two values of water flow rate, *Q*, were applied (*Q* = 0.5 and 1 L/min). The value of the copper head surface of *S* = 4.91 cm^2^ and the physical data for water *c*_p_ = 4.187259 J/(g K) and *ρ* = 998999.7 g/m^3^ were taken for calculations. The *SG-100* torch with the anode 03083-175 and the cathode 03083-129 and the gas injector 03083-112, mounted on a 5-axis *ABB IRB-6* industrial robot was used throughout the experiments. The torch trajectory is shown in Figure 4. The trajectories were repeated three times in the experiments simulating the deposition of HA coatings and once in the experiments simulating the deposition of TiO_2_ coating. The suspension liquid (no solid charge was used), with a flow rate of 20 g/min and composed of H_2_O + 50 wt % C_2_H_5_OH, was introduced by an internal injector through a nozzle of 0.5 mm of internal diameter. The heat flux was determined in the experiments simulating the spray condition of HA as described in [6] as a fifth set of parameters and titanium oxide as described in [8] with the exception of spray distance, which was taken as 85 mm instead of 57 mm, which is used effectively to spray real coatings.

These spray variables are collected in Table 1. The working gas used throughout all the experiments was a mixture of 45 Standard liter per minute (slpm) of Ar and 5 slpm of H_2_. The investigations were made varying the following operational processing parameters: the power of the plasma torch, the spraying distance and the speed of the robot (to which the plasma torch was attached). 

**Table 1:** Plan of experiments for determining the heat flux input to the substrate.

Experiment number	Power input to plasma, kW	Distance between torch and substrate, mm	Linear speed of robot, mm/s	Cooling water flow rate, L/min	Suspension liquid used	Parameters simulating deposition of:
1	33	70	500	0.5	No	HA
2				1.0		
3				0.5	Yes	
4				1.0		
5	40	85	250	0.5	No	TiO_2_
6				1.0		
7				0.5	Yes	
8				1.0		

The temperature of incoming water (*T*_1_ in Figure 3b) was measured before each experiment. The temperature of outcoming water was measured continuously during the experiments. The temperature of the copper head was measured during the experiments with the use of an *IN 5* pyrometer from *Impac*.

## 3. Modeling of the Sintering of Fine Grains in the Coating during Deposition

**Figure 5:**
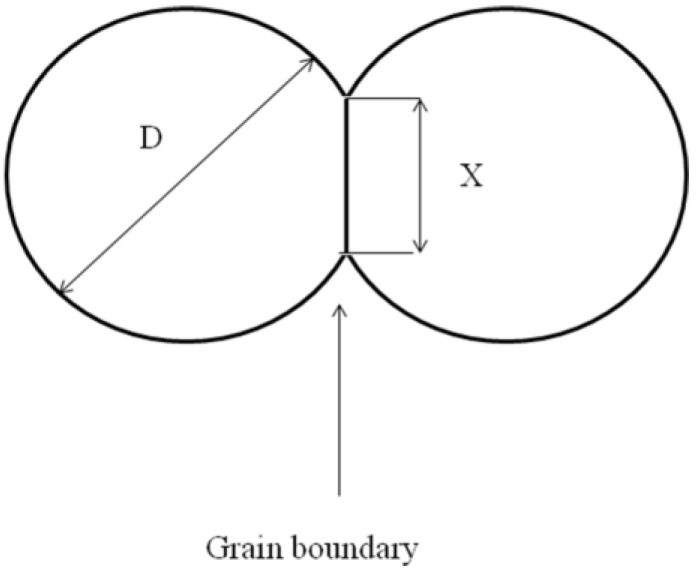
Sketch of a model of the sintering of two particles. *D* = cross-sectional area of the particle; *X* = neck size.

The small grains deposited on the substrate during the coating’s build-up are heated mainly by convective heat flux coming from the plasma jet. However, their temperature at impact may vary: a part of the small grains may remain at the periphery of the plasma jet and, independent of their thermal history (heating up, melting…), contact the substrate as a solid. Let us consider in detail the process of sintering of the particles adhering to the surface. According to the classical theory of sintering [9], one can distinguish four stages of this process: adhesion, initial stage, intermediate stage and final stage. Adhesion occurs almost immediately after mechanical contact between particles. So the starting point is an assembly of contacting particles. The initial stage of sintering corresponds to the period during which the inter-particle contact area increases from 0 to 0.2 of the cross-sectional area of the particle and neck size ratio *X*/*D* increases from 0 to 0.1 (Figure 5). The main driving force for sintering is the reduction of surface energy of the particles.

A few sintering models are available to provide an estimation of the neck’s growth rate for different mechanisms of matter transport: plastic flow, evaporation-condensation, volume diffusion, grain boundary diffusion and surface diffusion. The result can be summarized by the following expression of the neck size *vs.* sintering time *t* under isothermal conditions:

(2) $$X(t)=\frac{C^{1/n}(T)}{D^{m-n}}t^{1/n}$$

The mechanisms and corresponding values of the coefficients are summarized in Table 2. This equation can be reformulated to show the time required for the growth of neck diameter up to the value *X*:

(3) $$t(X)=\frac{D^{m}}{C(T)}{\left(\frac{X}{D}\right)}^{n}$$

In temperatures below half of the melting point, only the three mechanisms listed in Table 2 contribute significantly to the sintering time, while others are negligible. The material data needed to calculate the sintering time are collected in Table 3. 

**Table 2:** Initial-stage sintering equations [9].

No	Mechanism of matter transport	The values of coefficients in equations (2) and (3)		
		*n*	*m*	*C*(*T*)
1	Volume diffusion	5	3	$80D_{v}\gamma \Omega /kT$
2	Grain boundary diffusion	6	4	$20\delta_{b}D_{b}\gamma \Omega /kT$
3	Surface diffusion	7	4	$56\delta_{s}D_{s}\gamma \Omega /kT$

**Table 3:** Numerical data used for calculation of sintering time.

Parameters	HA		TiO_2_	
	Numerical data	Commentary and reference	Numerical data	Commentary and reference
*θ*, K/s	0.17	[18]	-	-
*D*, m	6 × 10^-8^	[18]	-	-
*D*_v0_, m^2^/s	5 × 10^-11^	Estimated from fitting, [18]	2 × 10^-7^	Oxygen diffusivity was taken, [14]
*δ*_b_*D*_b0_, m^3^/s	4 × 10^-21^	Calculated, [10]	6.3 × 10^-18^	Calculated, [10]
*δ*_s_*D*_s0_, m^3^/s				
*Q*_v_, J/mol	1.4 × 10^5^	Estimated from fitting, [18]	2.51 × 10^5^	Oxygen diffusivity was taken, [14]
*Q*_b_, J/mol	8.4 × 10^4^	Calculated, [10]	1.51 × 10^5^	Calculated, [10]
*Q*_s_, J/mol				
*γ*, J/m^2^	4.67 × 10^-2^	[12]	0.7	[15]
*Ω*, m^3^	5.28 × 10^-28^	[11]	6.24 × 10^-29^	[16]

There were many available data concerning TiO_2_, but much less for HA. Some coefficients, for this material, were approximated. These approximations are described in more detail in the Results section.

## 4. Results

### 4.1. Calorimetric determination of heat flux

**Figure 6:**
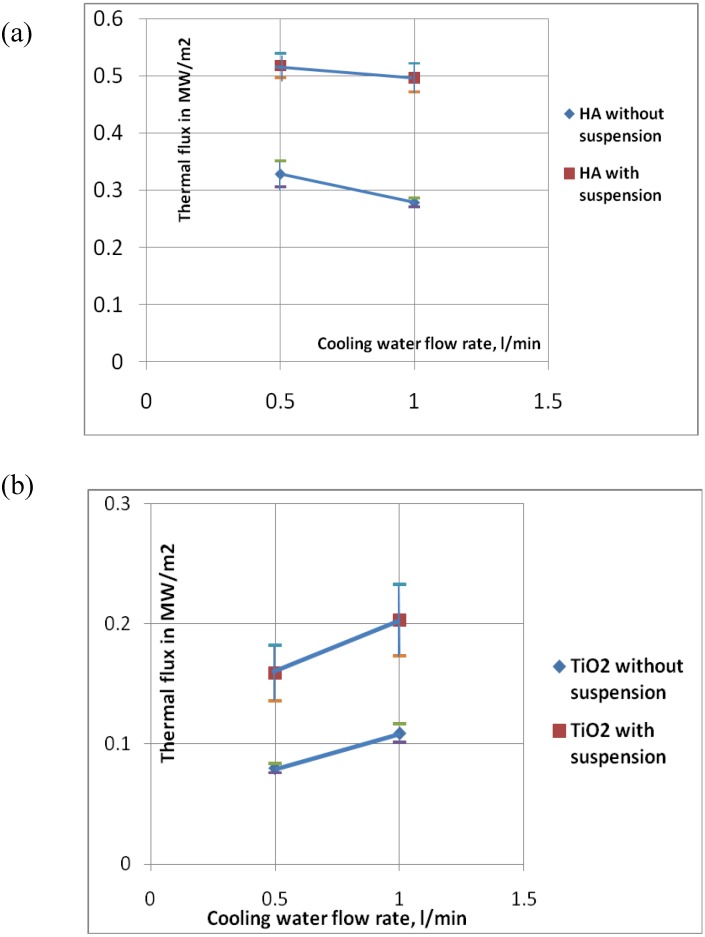
Thermal fluxes heating copper head *vs.* cooling water flow rates measured during calorimetric measurements simulating deposition of HA coatings (a), and TiO_2_ coatings (b).

**Figure 7:**
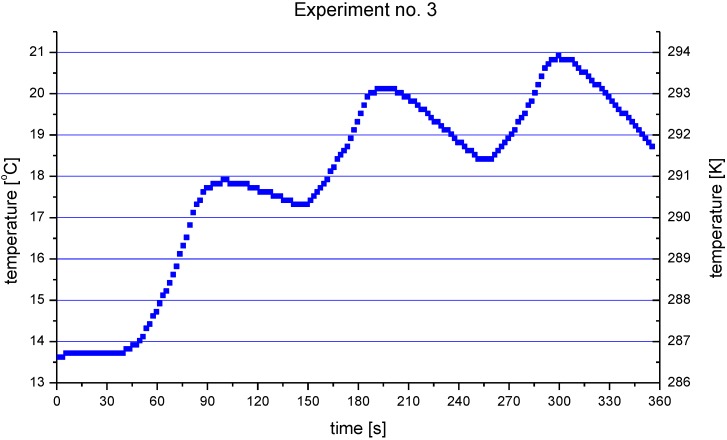
Outlet cooling water temperature in the experiment no. 3 simulating the deposition of HA with the use of the water flow rate of *Q* = 0.5 L/min.

The heat fluxes measured in all experiments are shown in Figure 6 and the temperatures of the copper head surface during these measurements are collected in Table 4. An important point is that the fluxes depend slightly on the cooling water flow rate. A greater cooling water flow rate results in lower values of heat flux for the conditions simulating the deposition of HA, *i.e.*, short spray distance and high torch linear velocity. The tendency is inversed for the condition simulating the deposition of TiO_2_. This effect must have been related to more intensive cooling of the copper head at greater water flow rate. The maximum temperatures of the surface of the copper head ranged from *T* = 423 K to *T* = 486 K. Knowing that copper conducts heat very well, it means that the cooling water, which was in contact with the substrate, must have partly evaporated. The vapors were then mixed with water, for which the outlet temperature was no greater than 294 K, as shown by the evolution of temperature measured for the greatest heat flux conditions in the experiment no. 3, which is shown in Figure 7. The dependence of the measured heat flux on cooling water flow rate could have been related to the mixing of the water vapors with water and resulting condensation. The condensation may have been incomplete at the thermocouple measuring outlet temperature in some experimental conditions. Finally, the exchange of heat between the cooling water and thermocouple could have depended on its flow rate. Heat flux absorbed by a substrate at the condition simulating the deposition of TiO_2_ is about *q* ≈ 0.08 MW/m^2^ without suspension. This value is about three-times lower than the heat flux measured for HA spray conditions, which might result from the great spray distance of 85 mm used in the experiments. The application of suspension liquid increases the heat flux two-times, up to about *q* ≈ 0.16 MW/m^2^ (Figure 6b).

**Table 4:** Temperatures of the copper head during the calorimetric heat flux measurements.

Experiment no.	Parameters simulating deposition of:	Maximum temperature of copper head, K
1	HA	427
2		443
3		458
4		486
5	TiO_2_	423
6		430
7		446
8		434

The heat flux absorbed by a substrate at the condition simulating the deposition of HA, is equal to about *q* ≈ 0.30 MW/m^2^ without suspension and *q* ≈ 0.51 MW/m^2^ with suspension (Figure 6a). This increase of about 70% results from the energy liberated from the burning of ethanol included in suspension liquid in air.

### 4.2. Theoretical analysis of TiO_2_ sintering

When considering diffusion in single component materials, one can take into account one value of the vacancy volume and one value of diffusivity, which is, in fact, self-diffusivity. However, for the ionic ceramics as TiO_2_, both the Ti^+^ cations and O^-^ anions diffuse in the stoichiometric proportion. Generally, for a ceramic A_α_B_β_, one has to consider the effective values of atomic volume and diffusivity as shown in the following equations [10]:

(4) $$D_{eff}=\frac{\left(\alpha +\beta \right)D_{A}D_{B}}{\beta D_{A}+\alpha D_{B}}$$

and

(5) $$\Omega_{\mathrm{eff}}=\frac{\Omega }{\alpha +\beta }$$

In Equation (5), Ω is the atomic volume (unit cell volume) of the A_α_B_β_. The above equations are based on the hypothesis that the difference in diffusivities between cations and anions is small. In the case of the difference between the atoms being significant, e.g., *D*_A_ >> *D*_B_, the effective diffusivity is dominated by the slower species, then $D_{e\text{ff}}≅D_{B}$ and $\Omega_{\text{eff}}=\Omega /\beta$ [10].

**Figure 8:**
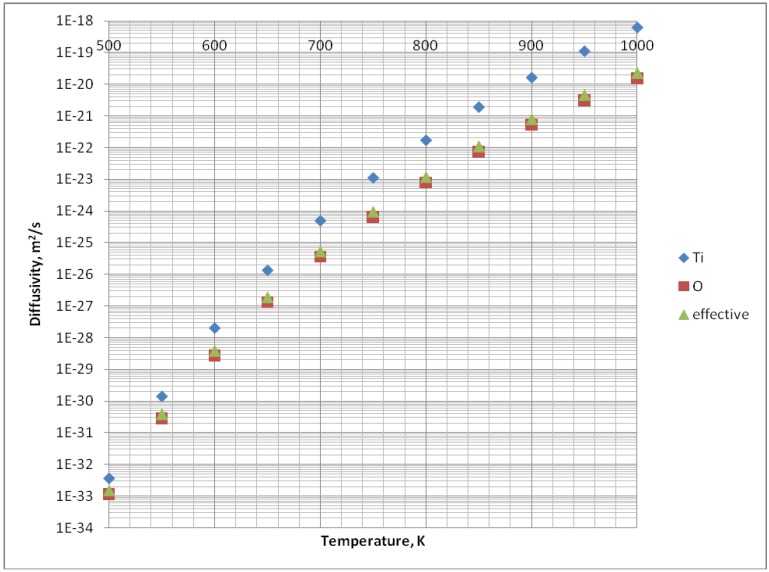
Diffusivity of Ti^+^ and O^-^ in TiO_2_ and effective diffusivity of mass-transport *D_eff_* *vs.* temperature (see the explanation in the text).

The volume diffusion data for TiO_2_ were taken from Samsonov [14] and are shown in Table 3. The effective diffusivity was calculated from Equation (4) by taking *α* = 1 and *β* = 2, and is shown in Figure 8.

It is clear that the effective diffusivity is close to the diffusivity of oxygen. One may conclude that the transport of mass is controlled by oxygen. Consequently, the oxygen diffusivity may be used in calculations of effective diffusivity for mass-transport and the effective atomic volume is equal to $\Omega_{\text{eff}}=\Omega /2$.

**Figure 9:**
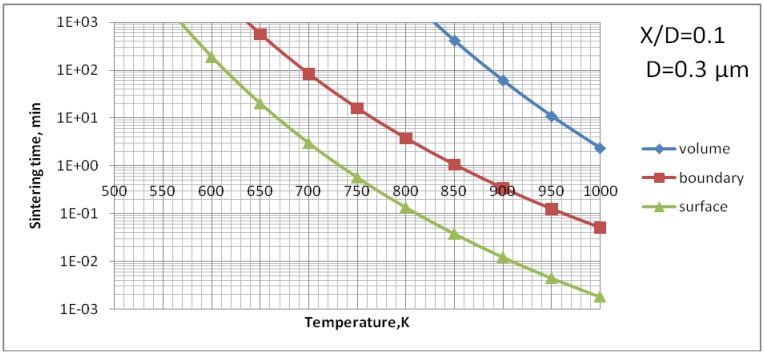
Sintering time of TiO_2_ particles of an initial diameter of *D* = 0.3 µm *vs.* temperature for three mechanisms of mass-transport: volume, grain boundary and surface diffusion.

The data for grain boundary diffusion and surface diffusions could not be found. Their evaluation was made by using the phenomenological relations proposed by Frost and Ashby [10], namely, $\delta_{b}D_{b0}=\delta_{s}D_{s0}=0.1\Omega^{1/3}D_{v0}$ and $Q_{b}=Q_{s}=0.6Q_{v}$. Figure 9 shows the results of calculations of all three possible mechanism of sintering for the size of TiO_2_ by supposing that the size of sintered particles is equal to the mean value of particles used to formulate the suspension for plasma spray, *i.e.,* *D* = 0.3 µm [3,8]. 

**Figure 10:**
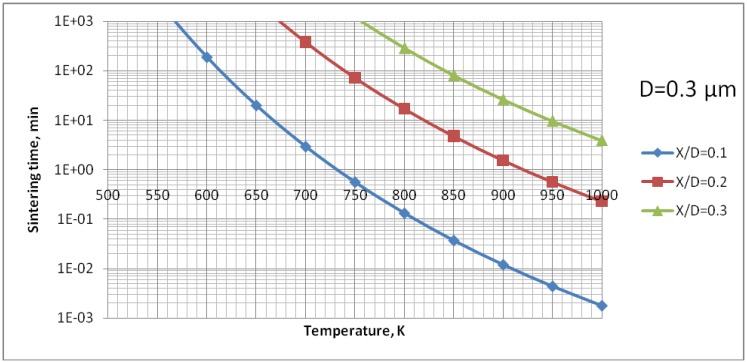
Sintering time of TiO_2_ particles having initial diameter of 0.3 µm to reach the necks diameter ranging from *X/D* = 0 to *X/D* = 0.1; 0.2 or 0.3 *vs.* temperature for surface diffusion mechanism of mass-transport.

**Figure 11:**
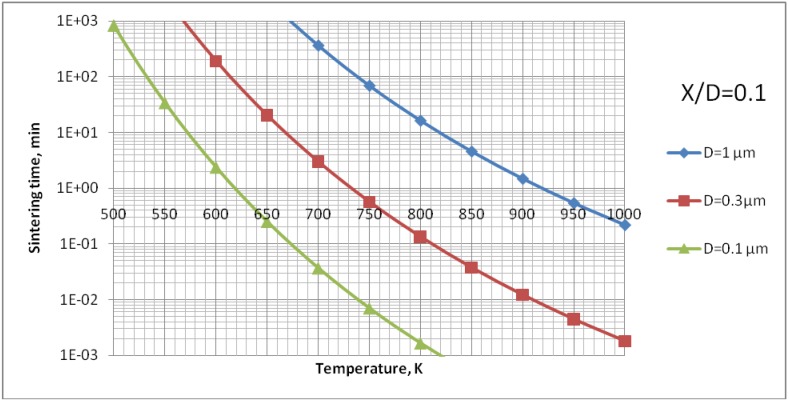
Sintering time of TiO_2_ with an initial diameter of *D* = 0.1; 0.3 or 1 μm to reach the neck size of *X*/*D* = 0.1 *vs.* temperature for surface diffusion mechanism (case of suspension plasma spraying with an internal injector).

The analysis of Figure 9 enables us to find out that the surface diffusion mechanism is predominant. Subsequently, the calculations for this mechanism were realized to find out the time necessary to achieve different sizes of neck ratios at different temperatures (Figure 10). Finally, Figure 11 shows the calculations for different sizes of titania particles. This calculation corresponds to the suspension plasma spray experiments made with internal injection, in which the particles become agglomerated and molten in the plasma jet as shown in Figure 2b.

### 4.3. Theoretical analysis of HA sintering

The data concerning volume, grain boundary and surface diffusion for HA could not be found in the textbooks [9,17]. The estimation of effective diffusivity was made with the use of the experimental data regarding shrinkage of this material. The linear shrinkage is related to the neck size by a simple equation [9]:

(6) $$\varepsilon =\frac{1}{4}{\left(\frac{X}{2D}\right)}^{2}$$

The negative sign of shrinkage is ignored. By combing the Equations (3) and (6), one can obtain the shrinkage as a function of time *ε*(*t*) and shrinkage rate $\dot{\varepsilon }(t)$, supposing the initial stage of sintering in isothermal conditions:

(7) $$\varepsilon (t)=\frac{1}{4}{\left(\frac{C(T)t}{D^{m}}\right)}^{2/n}$$

(8) $$\dot{\varepsilon }(t)=\frac{1}{2n}{\left(\frac{C(T)}{D^{m}}\right)}^{2/n}t^{\frac{2}{n}-1}$$

It has to be underlined that the surface diffusion mechanism produces neck growth without any shrinkage [9]. In this case, the kinetic coefficient in Equation (7) is equal to *C* = 0. 

Equations (7) and (8) are valid under isothermal conditions. However, the initial stage of shrinkage is practically impossible to investigate at a constant, high temperature because of difficult control over the phenomena that can happen during the preheating. It is easier to carry out an experiment with a constant heating rate *θ*, starting from an initial temperature *T*_0_ as done by Jokanovic *et al.* [18]. For small *θ*, it is possible to replace *t* by (*T*-*T*_0_)/θ in Equation (8), and to calculate the shrinkage as a function of temperature:

(9) $$\varepsilon (T)=\frac{1}{2n{\left(D^{m}\theta \right)}^{\frac{2}{n}}}\int_{T_{0}}^{T}C^{\frac{2}{n}}(\tilde{T}){\left(\tilde{T}-T_{0}\right)}^{\frac{2}{n}-1}d\tilde{T}$$

**Figure 12:**
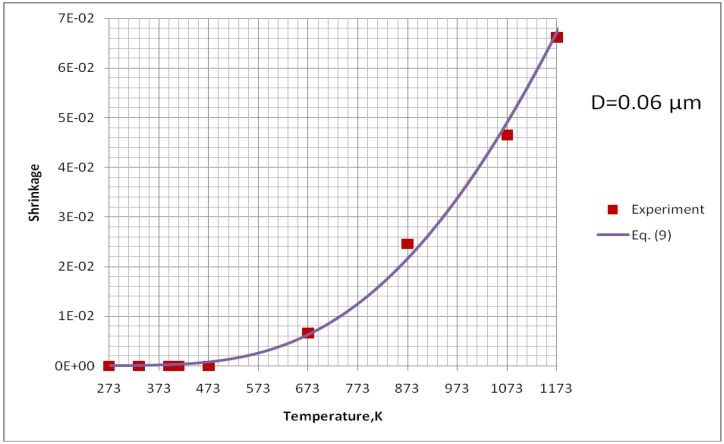
Fitting of experimental points obtained for shrinkage of HA at constant heating rate, *θ* = 0.17 K/s, by Jokanovic *et al.* [18] to Equation (9).

**Figure 13:**
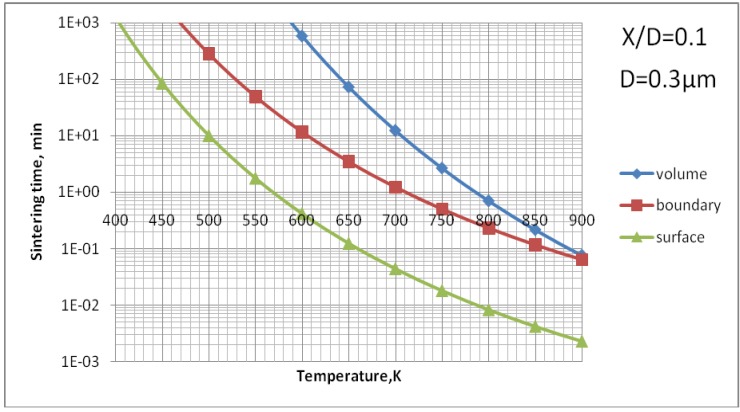
Sintering time of HA particles of an initial diameter of *D* = 0.3 µm *vs.* temperature for three mechanisms of mass-transport: volume, grain boundary and surface diffusion.

**Figure 14:**
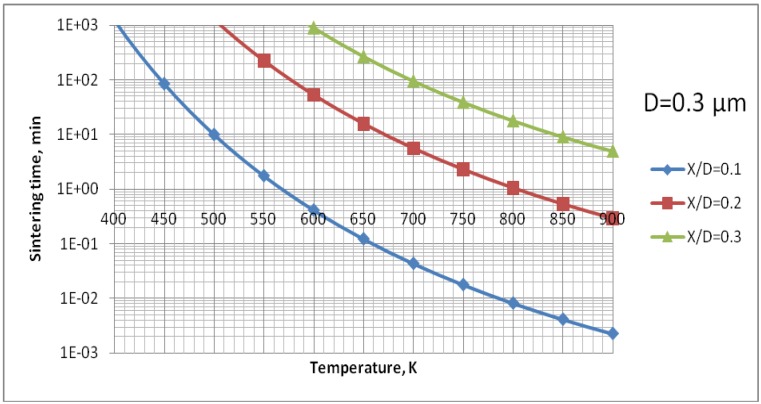
Sintering time of HA particles having an initial diameter of 0.3 µm to reach the necks diameter ranging from *X/D* = 0 to *X/D* = 0.1; 0.2 or 0.3 *vs.* temperature for surface diffusion mechanism of mass-transport.

**Figure 15:**
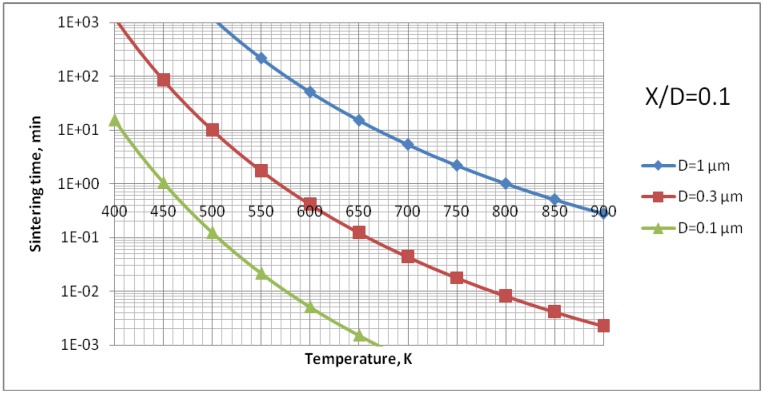
Sintering time of HA particles of initial diameter of *D* = 0.1; 0.3 or 1 μm to reach the neck size of *X*/*D* = 0.1 *vs.* temperature for surface diffusion mechanism (case of suspension plasma spraying with an internal injector).

This equation was used to determine the effective diffusivity from the experimental data obtained by Jokanovic *et al.* [18] with the heat rate of *θ* = 0.17 K/s. The experimental points were fitted to the curve given by Equation (9) as is shown in Figure 12. The fitting was optimized for two parameters, namely, *D*_b0_ and *Q*_b_. All parameters necessary to compute the sintering time of HA are collected in Table 4. The results of calculations for all three possible mechanisms of sintering for the HA particle initial size of *D* = 0.3 µm are shown in Figure 13. This value corresponds to the small particulates visible on the surface of coatings’ sprayed with the use of continuous-stream injectors, for which a typical example is shown in Figure 2a. The analysis of Figure 13 enables to find out that, similarly to the calculation for TiO_2_, the surface diffusion mechanism is predominant. Subsequently, the calculations for this mechanism were realized to find out the time necessary to achieve different sizes ratios at different temperatures (Figure 14). Finally, Figure 15 shows the calculations for different sizes of hydroxyapatite particles. This calculation corresponds to different sizes of particles in the coatings obtained in the suspension plasma spray experiments made with continuous-stream internal injection in which the particles agglomerate and become molten in the plasma jet as shown in Figure 2a.

## 5. Discussion

**Figure 16:**
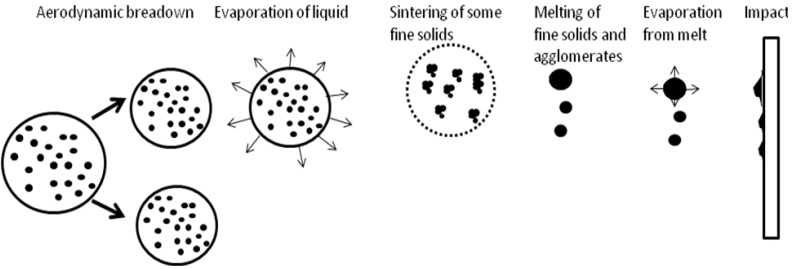
Evolution of a suspension droplet in the high temperature plasma or flame [1].

The microstructure of thermally sprayed deposits results mainly from the state of the particles arriving on the substrate or on the previously deposited coating. The conventional thermal spray process generally uses powders as the feedstock, and the particles arriving on the substrate can be molten, partly molten or unmolten, depending on their trajectory in flame or jet [19]. The use of suspension as the feedstock to spray renders the phenomena in-flight more complicated. The droplets of suspension, depending on their trajectory in flame or jet, can be submitted to the primary and secondary break-up, liquid evaporation, fine solids agglomeration, melting (see Figure 16).

**Figure 17:**
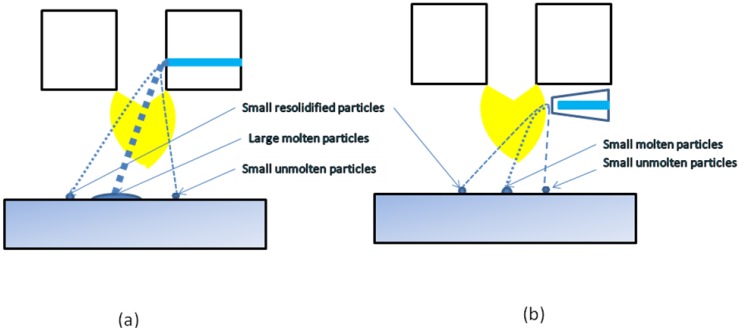
Possible trajectories of droplets/particles during plasma spraying with the use of internal, continuous-stream injection (a) and external, atomizing injection (b).

Supposing radial injection of suspension, the trajectory of droplets can be [1,20]: (i) outside of the jet or flame, and the solids contained in such droplets (which do not penetrate into plasma jet) arrive unmolten on the substrate and correspond to the solids used to formulate the suspension visualized in Figure 1 (as the irregular particles) or in Figure 2 a and b (as agglomerated zones); (ii) in the center of the jet or flame, and these particles will be, at internal continuous-stream injection of suspension, agglomerated and well molten on impact with the substrate as shown in the dense areas in Figure 2a and b; (iii) traversing the jet or flame, and the particles having such trajectory are re-solidified on arriving on the substrate, such as the round grains shown in Figure 1. The sketch of possible trajectories is shown in Figure 17. The energy flux arriving to the substrate heats up the growing coating. The resulting temperature depends on the operational conditions of spraying (Table 3). Among the spray parameters, the spray distance most influences the coating’s temperature. The heat flux values found in the present study are slightly lower than that of the convective flux equal to *q* = 1.1 MW/m^2^ found by Marynowski *et al.* [21] for the spray distance of 75 mm and Ar+H_2_ plasma supplied with 30 kW of electric power. The flux estimated by Tingaud *et al.* [24] was even greater, *q* = 30 MW/m^2^, for Ar+H_2_ plasma. The data of the latter study were originally obtained in the PhD thesis of Etchart-Salas [23], who used the spray distance of 40 mm and torch in rest with regard to substrate. The values, being in the range of *q* = 0.1 – 0.5 MW/m^2^, were obtained in the present study with the torch being in movement and the experiment was designed to simulate the flux obtained at the coating deposition.

**Figure 18:**
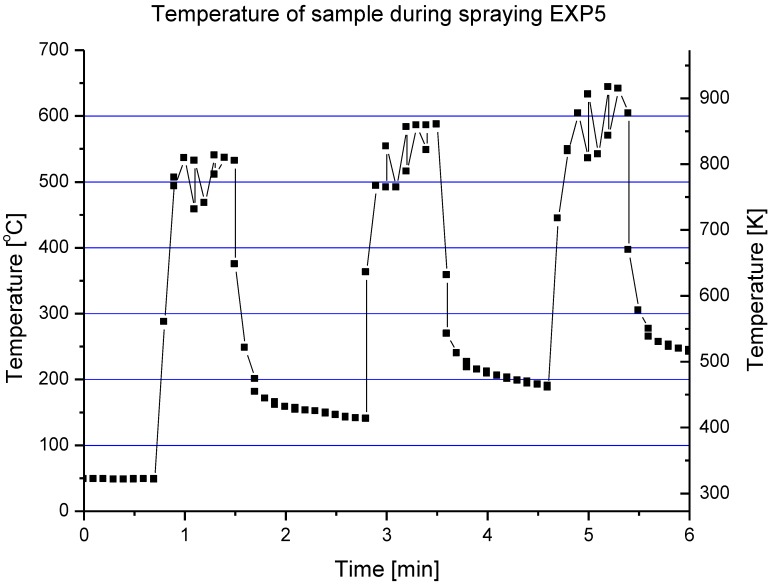
Evolution of a typical surface temperature during HA coating deposition [7].

The processes of the spraying of TiO_2_ and HA coatings, described in this paper, were carried out in such a way that the torch realized the scans over the substrate. One trajectory over a substrate lasted about 10 s. After five such trajectories the torch was put in rest until the coating cools down to the temperature of about *T* = 303 K. The resulting temperature cycles had a duration of about 1 min and minimum temperatures of about 303 K and maximum temperatures reached 663 K for TiO_2_ (sprayed using internal continuous-stream injector) and about 873 K for HA as shown in Figure 18. Among the different sintering mechanisms, only the surface diffusion method seems to be able to result in the sintering of TiO_2_ or HA grains in such temperature-time conditions as show it Figure 9 and Figure 13, respectively.

**Figure 19:**
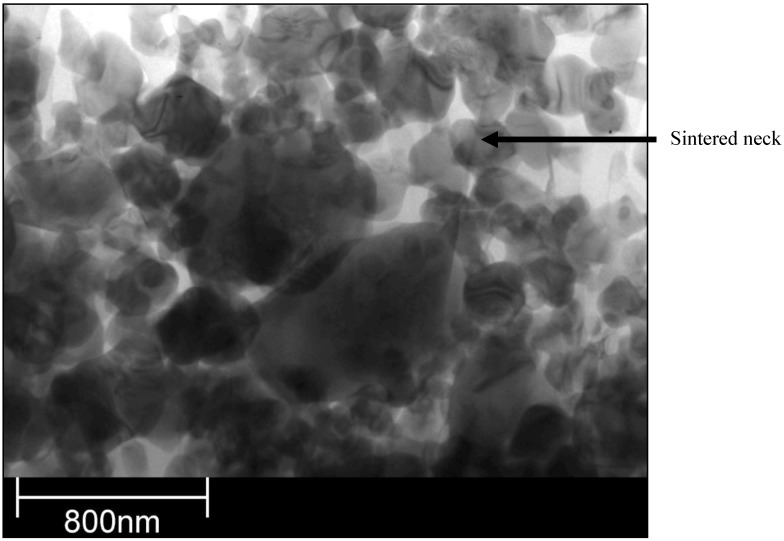
Transmission electron micrograph of an agglomerated zone of HA coating obtained by suspension plasma spraying [7].

The sintering of TiO_2_ grains having a size of 0.3 µm would result in formation of a neck having a size of about *X*/*D* = 0.1 (Figure 10) for the experimental conditions characterized by temperature cycle of 650 K during one minute. Necks of greater size would need much longer times to grow. The cycle for HA (873 K during 1 min) would enable a neck of the size of about *X*/*D* = 0.2 to develop (Figure 14). The heat flux coming from plasma jet and burning suspension, and resulting thereof coating temperature, would result in the sintering of hydroxyapatite grains. Finally, the start of the sintering processes, *i.e.* the development of the neck of *X*/*D* = 0.1, depends very strongly on the size of the particles. Two TiO_2_ particles having diameter of 0.1 µm would sinter such a neck at the temperature of *T* = 650 K in a time as short as 12 s (Figure 11). A HA particle of such size would develop such a neck at the experimental temperature of *T* = 873 K within milliseconds (see Figure 15). Such sintering seems to be confirmed by the transmission electron microscope observations of suspension plasma sprayed HA coatings observed by Podlesak *et al.* [7] and shown in Figure 19.

The last point that should be stressed is the presence of vapors of water and of ethanol (the part of ethanol which did not burn out) from the suspension spraying atmosphere around the growing coating. Such atmosphere would rather promote sintering.

## 6. Conclusions 

Suspension plasma spraying is a process which enables the production of finely grained nanometric or submicrometric coatings. The suspensions are formulated with the use of fine powder particles in water or alcohol. Subsequently, the suspension is injected into the plasma jet and the liquid additives evaporate. The remaining fine solids are molten and agglomerate with other molten particles or remain solid, depending on their trajectory in the plasma jet. The coating’s microstructure results from these two groups of particles arriving on a substrate. The previous experimental studies carried out for plasma sprayed titanium oxide and hydroxyapatite coatings enabled us to find out, depending on the suspension injection mode, a microstructure composed of fine grains or a microstructure characterized by two zones: (i) a dense zone formed by well molten particles, and, (ii) an agglomerated zone formed by fine solid particles that arrived on the substrate in a solid state.

The present paper concentrates on the theoretical analysis of the process of formation of finely grained microstructure, which corresponds also to the agglomerated zone. The experimental section deals with the calorimetric measurements of the convective heat flux input to the substrate in the experimental conditions simulating the coating deposition. The heat flux was found to be in the range from 0.08 to 0.5 MW/m^2^, depending on experimental conditions. The theoretical section of the paper deals with the calculation of possible sintering of fine particles arriving on the substrate in the solid state. The models of volume, grain boundary and surface diffusion sintering were analyzed and adapted to the size and chemical composition of particles, temperature and time scales corresponding to suspension plasma spraying conditions. The model of surface diffusion was the most appropriate to describe the sintering at suspension plasma spraying. It was found that it is possible to develop necks having the relative size of 10% of particle diameter during the thermal cycles occurring at the processing of sprayed coatings. Consequently, the sintering of the fine grains deposited on the substrate was proved to occur in the experimental conditions of suspension plasma sprayed TiO_2_ and HA coatings’ growth. The transmission electron microscopic observations of the agglomerated zone in the HA coating obtained by using such a technique confirms this conclusion. Further research should characterize the local mechanical properties of agglomerate zones by means of nanoindentation tests and compare the properties of such agglomerate zones to those of dense zones. 

## Symbols and Acronyms

All units are SI unless otherwise stated in the text

*A*: symbol of element
*B*: symbol of element
*C*: kinetic coefficient
*c*_p_: specific heat at constant pressure
*D*: particle diameter
*D*_A_: diffusivity of element A
*D*_b_: grain boundary diffusivity, *D*_b_ = *D*_b0_ exp[-*Q*_b_/(RT)]
*D*_b0_: grain boundary diffusion factor
*D*_eff_: effective diffusivity
*D*_s_: surface diffusivity, *D*_s_ = *D*_s0_ [-*Q*_s_/(RT)]
*D*_s0_: surface diffusion factor
*D*_v_: volume diffusivity, *D*_v_ = *D*_v0_ exp[-*Q*_v_/(RT)]
*D*_v0_: volume diffusion factor
HA: hydroxyapatite
*k*: Boltzmann constant, *k* = 1.38 × 10^-23^ J/K
*M*: molecular mass
*m*: constant in Equation 2 and 3
*n*: constant in Equation 2 and 3
*Q*: water flow rate
*Q*_b_: grain boundary diffusion activation energy, *Q*_b_ = 0.6*Q*_v_
*Q*_s_: surface diffusion activation energy, *Q*_s_ = 0.6*Q*_v_
*Q*_v_: volume diffusion activation energy
*q*: heat flux
*R*: universal gas constant, *R* = 8.314 J/(mol K)
*S*: surface
SEM: scanning electron microscope
*T*: temperature
*T*_0_: initial temperature
*t*: time
*X*: neck diameter
*X*/*D*: neck size ratio
*α*: stoichiometric index
*β*: stoichiometric index
*γ*: surface energy
*δ*_b_: width of grain grain boundary diffusion zone, *δ*_b_ = 0.1*Ω*^1/3^
*δ_s_*: depth of surface diffusion zone, *δ_s_* = 0.1*Ω*^1/3^
*ε*: linear shrinkage
*θ*: heating rate
*ρ*: density
Δ: difference
*Ω*: atomic volume
*Ω*_eff_: effective atomic volume
